# Education and Implementation Co-Design Approaches for READI-SET-GO using CFIR Framework

**DOI:** 10.1093/geroni/igaf122.2319

**Published:** 2025-12-31

**Authors:** Zahra Karimi, Priyanka Shrestha, Jessica Fleming, Erica Husser, Donna Fick, Edward Marcantonio, Chia Jou Lin

**Affiliations:** The Pennsylvania State University, University Park, Pennsylvania, United States; The George Washington University, Washington, District of Columbia, United States; The Pennsylvania State University, University Park, Pennsylvania, United States; The Pennsylvania State University, University Park, Pennsylvania, United States; The Pennsylvania State University, University Park, Pennsylvania, United States; Beth Israel Deaconess Medical Center, Boston, Massachusetts, United States; Taipei Medical University, Taipei City, Taipei, Taiwan (Republic of China)

## Abstract

**Background:**

Delirium in hospitalized older adults can lead to severe outcomes, including falls, antipsychotic use, and misdiagnosis as Alzheimer’s disease, contributing to cognitive decline and mortality. Early detection is critical, yet implementation barriers include lack of clinical prioritization, competing demands, time constraints, discomfort with screening tools, and limited leadership support. This study explores education and implementation strategies for integrating the Ultra-Brief Confusion Assessment Method (UB-CAM) into the electronic health record (EHR) to enhance delirium screening and management.

**Method:**

Using a stepped-wedge design across three hospitals over 42 months, nurses conduct daily UB-CAM screenings, with new units launching every six months. Implementation strategies are guided by RE-AIM and CFIR frameworks and include: (1) EHR-integrated app-based screening, (2) stakeholder engagement, (3) co-designed education (in-person and online), (4) hands-on case-based practice with feedback, and (5) addressing organizational factors such as leadership, culture, and workflow alignment.

**Results:**

In the first intervention block, 100% of eligible nurses completed the education program (65% in-person, 35% online), utilizing video-based case studies for hands-on learning and feedback. We describe champion selection, use of screening feedback reports, leadership engagement, and co-design of clinical strategies to enhance adherence.

**Discussion:**

UB-CAM integration into EHR, combined with co-designed education and multifaceted implementation strategies, has improved engagement and adherence. Hybrid training, technology-driven formats, and organizational leadership involvement have optimized adoption. Future research should assess the sustainability of these approaches for delirium screening.

